# A Study of Dosimetric Feasibility of a New Approach of Radiosurgery for Multiple Sclerosis-Related Trigeminal Neuralgia

**DOI:** 10.7759/cureus.91411

**Published:** 2025-09-01

**Authors:** Ajay Zheng, Juying Chen, Xiaodong Wu, Aizik L Wolf

**Affiliations:** 1 Radiation Oncology, Miami Neuroscience Center, Larkin Community Hospital, Miami, USA; 2 Medical Physics, New York Proton Center, New York, USA; 3 General Surgery, Fujian Provincial Hospital, Provincial Clinical Medical College of Fujian Medical University, Fuzhou, CHN; 4 Medical Physics, Executive Medical Physics Associates, Miami, USA; 5 Neurological Surgery, Miami Neuroscience Center, Larkin Community Hospital, Miami, USA

**Keywords:** ms-tn, multiple sclerosis, radiation-induced toxicity, radiosurgery, trigeminal neuralgia

## Abstract

Background/objectives: Multiple sclerosis-related trigeminal neuralgia (MS-TN) presents challenges due to multifocal demyelination and diffuse neural involvement. Conventional single-isocenter gamma knife stereotactic radiosurgery (SRS) treats approximately 6 mm of the trigeminal nerve, which is often inadequate for MS-TN. This study evaluated the feasibility of a double-isocenter technique to expand nerve coverage to 10 mm while maintaining brainstem dose limits.

Methods: A dosimetric analysis was conducted on 10 patients previously treated for trigeminal neuralgia (TN) using conventional SRS. The double-isocenter configuration involved manually positioning two isocenters of a 4 mm gamma knife collimator, with an average separation of 4.8 mm, to create a consistent dose distribution covering a 10 mm segment of the trigeminal nerve. The treatment was prescribed to the 50% isodose line. To compare the single- and double-isocenter plans, dosimetric parameters including geometric nerve coverage, maximum brainstem dose (Dmax), and brainstem volume receiving ≥10 Gy (V(10Gy)) were analyzed.

Results: Replacing the single-isocenter with a double-isocenter configuration increased the prescription dose coverage along the trigeminal nerve from approximately 6.0 mm to 10.0 mm. Although the width of the 50% prescription isodose line perpendicular to the nerve axis slightly decreased from 5.9 ± 0.3 mm to 5.6 ± 0.5 mm, it remained sufficient to encompass the trigeminal nerve, which typically measures 2 mm to 3 mm in diameter. Similarly, the higher isodose lines, particularly the 80%, became slightly narrower, decreasing from 3.8 ± 0.2 mm to 3.1 ± 0.2 mm. This narrowing was effectively offset by an increased axial coverage along the nerve, from 4.0 ± 0.2 mm to 6.2 ± 0.8 mm. The mean brainstem Dmax was 23.4±2.9 Gy for single-isocenter and 22.5±3.3 Gy for double-isocenter plans (p=0.115), showing no statistically significant difference. The mean brainstem V(10Gy) increased from 0.054±0.011 cc (single-isocenter) to 0.079±0.024 cc (double-isocenter) (p<0.003).

Conclusions: The double-isocenter technique effectively expanded nerve coverage without compromising brainstem safety, as the brainstem Dmax remained within acceptable limits. Although the increase in brainstem V(10Gy) was statistically significant, its absolute value remained well below established tolerance guidelines, suggesting that the clinical impact is likely negligible. This approach demonstrates feasibility and supports future clinical trials to evaluate efficacy and long-term outcomes in MS-TN patients.

## Introduction

Trigeminal neuralgia (TN) is a neurological disorder marked by sudden, intense episodes of facial pain, often described as an electric shock-like, lancinating sensation that can severely impact quality of life [1,2]. The pain is typically unilateral, confined to the distribution of one or more branches of the trigeminal nerve, and triggered by innocuous stimuli such as touching the face, chewing, or speaking. Epidemiologically, TN affects approximately four to 13 per 100,000 people annually, with a higher incidence in women and individuals over 50 years old, influenced by a combination of genetic predispositions and environmental factors [1,3]. Pathogenesis primarily involves neurovascular compression at the trigeminal root entry zone, leading to demyelination and ectopic firing of nerve fibers, though idiopathic cases and secondary forms due to underlying conditions like tumors or vascular malformations also occur [1,2].

Clinically, TN presents as paroxysmal attacks lasting seconds to minutes, with possible periods of remission, but some patients experience concomitant continuous pain, adding to diagnostic complexity [2,3]. Diagnosis relies on the International Classification of Headache Disorders (ICHD-3) criteria, distinguishing classical TN (due to neurovascular compression), secondary TN (attributable to an underlying disease), and idiopathic TN (no identifiable cause), often requiring neuroimaging to rule out structural abnormalities [1,2]. This condition presents additional unique challenges in patients with multiple sclerosis-related trigeminal neuralgia (MS-TN), where demyelination associated with MS is thought to exacerbate TN's pathophysiology [2].

In MS-TN, the widespread neural involvement and progressive nature of MS often render the pain more resistant to conventional treatments, significantly diminishing the patient's quality of life [4,3]. The prevalence of TN in MS patients is estimated at 1% to 6.6%, far higher than in the general population, and it may manifest bilaterally or with atypical features like prolonged background pain due to pontine plaques affecting the trigeminal pathways [2,3].

The standard treatment for TN typically begins with pharmacotherapy, most commonly anticonvulsants like carbamazepine. However, drug resistance is a frequent issue, particularly in MS-TN patients [5]. Surgical interventions, such as microvascular decompression, glycerol rhizotomy, and balloon compression, offer alternatives but carry risks due to their invasive nature and potential complications [6]. Stereotactic radiosurgery (SRS), a non-invasive option, has emerged as a promising approach. Nonetheless, the effectiveness of SRS in MS-TN is often compromised by the diffuse and multifocal nature of neural damage, leading to variable pain relief and higher recurrence rates [7]. In two large retrospective studies, patients with MS-TN experienced a significantly shorter median duration of Barrow Neurological Institute (BNI) IIIb or worse pain relief following initial gamma knife radiosurgery (GKRS) compared to those with classical TN (1.1 vs. 4.6 years, p < 0.001). However, this difference was not observed after repeat GKRS, where the durations were comparable (4.0 vs. 3.8 years, p = 0.93), though higher rates of facial numbness were associated with repeat GKRS in both classical and MS-TN cases [7,8]. These findings align with clinical observations at our institution [8].

Additional literature has further elucidated the role of SRS in this subgroup. Similarly, a retrospective review in 2018 found that SRS provided long-term pain control in about 40% of MS patients, with facial numbness as a common side effect but overall good tolerance [9]. A prospective series in 2014 of 43 MS-TN patients treated with GKRS demonstrated initial response rates of over 80%, with neurovascular compression predicting better sustained outcomes, suggesting patient selection criteria could enhance results [10]. Long-term outcomes from a 2024 study on GKRS showed adequate pain relief in 71.4% of cases, though recurrence occurred in 30%, underscoring the need for optimized targeting strategies [11]. Another 2024 retrospective study on repeat GKRS for recurrent TN in MS patients indicated that it may be used effectively to prolong the duration of pain reduction [12]. Finally, a 2020 comparison of SRS and radiofrequency ablation for MS-TN found similar efficacy but different side effect profiles, highlighting the need for tailored approaches [13].

The current SRS approach for TN involves targeting a 4 mm to 6 mm segment of the trigeminal nerve using a single isocenter with a 4 mm collimator, delivering 40 Gy [14]. For MS-TN, effective and durable treatment may require a more aggressive therapeutic approach than for classical TN. Two primary strategies exist to achieve this higher dose threshold: increasing the prescribed dose or expanding the volume of coverage. Given the heightened risk of toxicity with repeat GKRS, increasing the dose is not the optimal strategy [14]. This consideration points to the alternative approach of expanding the treatment volume. We hypothesize that expanding the SRS treatment zone along the trigeminal nerve could improve outcomes for MS-TN patients. The MS lesions are often distributed along the nerve, and a longer treatment segment may more effectively target multiple demyelinated areas [15,16]. Extending the radiation dose coverage may create a more robust barrier to pain signal transmission, potentially reducing pain recurrence [17].

Before initiating a clinical trial, we conducted a dosimetric analysis of the brainstem dose distribution to ensure safety. The most critical concern in treating TN, particularly when considering dose escalation or expanding the treatment volume, is the potential increase in toxicity for the brainstem. The SRS must balance achieving effective pain relief with minimizing the risk of radiation-induced complications, especially for critical structures such as the brainstem. Data from prior studies indicate a need to limit the brainstem's exposure to a maximum dose of 35 Gy, with no more than 0.5 cc receiving over 10 Gy [18-20]. This step was essential to ensure that the brainstem dose remains within the safe limits.

## Materials and methods

Since 1994, over 2400 patients with TN, including those with multiple sclerosis (MS), have been treated at our institution (Miami Neuroscience Center, Larkin Community Hospital; Miami, FL, USA) using the Leksell Gamma Knife Stereotactic Radiosurgery system (Elekta AB, Stockholm, SWE). This extensive clinical experience has provided a strong foundation for understanding the effectiveness and safety of SRS in managing TN, including complex cases like MS-TN. For this dosimetric evaluation, we retrospectively reviewed our institutional database of patients treated for TN. Ethical review and approval were waived by the Institutional Review Board of the Larkin Community Hospital due to the retrospective nature of the study and the absence of any associated risk to patients. Informed consent was not applicable for the same reasons.

With over years of clinical experience, our institution has established a standardized treatment protocol. For initial treatments, isocenters are consistently placed near the root entry zone (REZ) of the trigeminal nerve, with a conservative brainstem dose constraint of <30 Gy, stricter than the commonly accepted 35 Gy threshold, to enhance safety. In retreatment scenarios, the isocenter is placed anterior to the original, with the prescription isodose line abutting the previous one, typically using a 25% reduced dose. This protocol is uniformly applied to all TN cases, including those with MS-TN.

Accordingly, patients were not selected specifically from the MS-TN population. Instead, 10 patients (five right-sided and five left-sided TN) were randomly chosen from a more recent treatment period between October 31, 2023, and May 14, 2024, during which 53 TN patients underwent GKRS. The selected patients ranged in age from 56 to 86 years (mean: 68.8). Aside from requiring conventional single-isocenter GKRS for unilateral TN and the availability of complete dosimetric data, including high-resolution MRI for treatment planning, no additional inclusion criteria were applied. This approach was justified by the study’s objective of assessing brainstem dose metrics, given the anatomical consistency of the brainstem aside from age-related variation.

Treatment planning was performed with the Leksell GammaPlan software version 11.3.2 (Elekta AB), using pre-treatment MRI scans acquired on a 1.5 T MRI unit, including T1-magnetization prepared rapid gradient echo (MPRAGE) sequences and T2-constructive interference in steady state (CISS) sequences (slice thickness 1 mm) for nerve visualization and brainstem delineation. The cisternal segment of the trigeminal nerve was specified as the target volume by the neurosurgeon. For the conventional single-isocenter plan, a single 4 mm collimator shot was placed at the REZ of the trigeminal nerve, slightly distal to the brainstem to avoid overdosing the brainstem, delivering a prescription dose of 40 Gy to the 50% isodose line, normalized to the maximum dose point. Treatments were pin-fixation frame-based to ensure precise immobilization and targeting accuracy.

To implement the new treatment strategy, two isocenters (shots) using the 4 mm collimator were placed manually along the trigeminal nerve, spaced adequately (4.5 to 5.0 mm) to achieve a consistent dose distribution covering a 10 mm segment of the trigeminal nerve in the cerebrospinal fluid (CSF) space. For planning, sector blocking was allowed to reduce brainstem exposure. This expanded coverage aimed to irradiate a larger portion of the nerve, potentially targeting more diffuse or multifocal demyelinated areas common in MS-TN. The prescription dose for this treatment was set at 40 Gy, prescribed to the 50% isodose line, with a maximum point dose of 80 Gy.

Critical structures, including the brainstem, were contoured according to Radiation Therapy Oncology Group (RTOG) guidelines. Dose-volume histograms (DVHs) were generated for each plan. Two critical dosimetric parameters were analyzed for each treatment plan to evaluate potential risks to the brainstem: (1) maximum dose (Dmax) to the brainstem, defined as the highest point dose within the brainstem contour; and (2) volume receiving 10 Gy or greater, V(10Gy), calculated as the absolute volume (in cc) of brainstem receiving ≥10 Gy. These parameters were chosen based on established literature thresholds for preventing cranial neuropathy, with constraints set at Dmax <35 Gy and V(10Gy) <0.5 cc [18-20].

Both the conventional single-isocenter configuration (the original plan used for patient treatment) and the double-isocenter (expanded coverage) configuration were analyzed for each patient using the same imaging dataset and software, allowing for a direct paired comparison of the dosimetric impact of each approach. Statistical comparisons between the two configurations were performed using paired two-tailed t-tests (α = 0.05) in Microsoft Excel version 16.85 (Microsoft Corp., Redmond, WA, USA) to determine whether the expanded coverage approach would result in unacceptable increases in radiation dose to the brainstem and provide critical information for the safe implementation of this strategy in future clinical trials.

## Results

**Figure 1:**
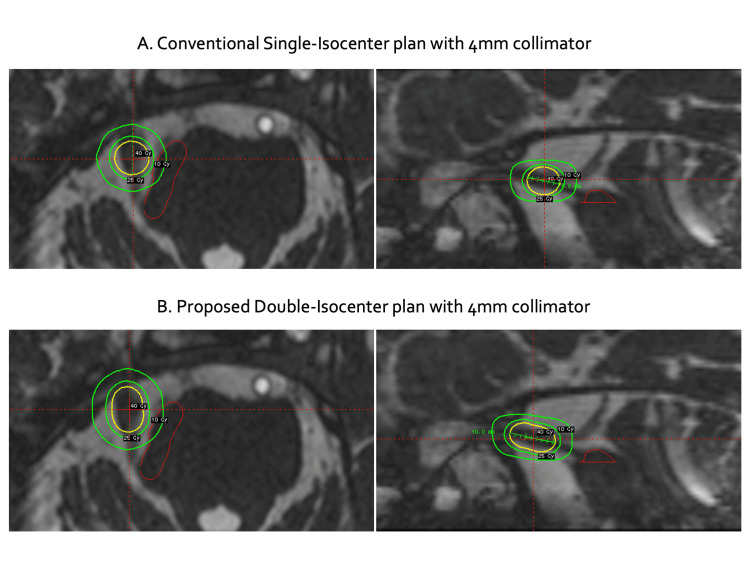
Dose distributions of the conventional single-isocenter plan (A) and the double-isocenter plan with 10 mm nerve segment coverage (B)

The dose distributions for single-isocenter and double-isocenter plans are illustrated in Figure 1, highlighting the expanded nerve coverage achieved by the double-isocenter configuration. The figure includes three isodose lines: 10 Gy, 25 Gy, and the prescribed dose of 40 Gy (yellow), superimposed on axial and sagittal MRI views to visualize the spatial relationship between the target (trigeminal nerve) and the adjacent brainstem. To minimize dose exposure to the brainstem, the expanded coverage was directed towards the trigeminal nerve ganglion, ensuring that the isodose fall-off was steepest in the direction of the brainstem.

The dosimetric metrics for the 10 cases are summarized in Table 1, which lists the covered nerve segment length, maximum brainstem dose (Dmax), and brainstem volume receiving ≥10 Gy (V(10Gy)) for both configurations. With 40 Gy prescribed to the 50% isodose line, the single-isocenter plan covered a mean nerve segment of 6.17 mm (range, 5.7-6.8 mm), representing the conventional approach. In contrast, the double-isocenter plan consistently extended the coverage to 10 mm, achieving a broader treatment zone along the nerve while preserving plan conformity and a steep dose gradient.

**Table 1:** Dosimetric metrics comparing single-isocenter and double-Iisocenter plans

Case ID	Site	Single-isocenter			Double-isocenter		
		Covered segment (mm)	Brainstem Dmax (Gy)	Brainstem V(10Gy) cc	Covered segment (mm)	Brainstem Dmax (Gy)	Brainstem V(10Gy) cc
1	Right	6.6	24.9	0.042	10.0	22.6	0.101
2	Right	5.8	21.9	0.052	10.0	21.4	0.088
3	Right	6.8	29.6	0.077	10.0	29.2	0.113
4	Right	6.4	25.5	0.057	10.0	24.4	0.101
5	Right	6.0	20.2	0.044	10.0	17.7	0.050
6	Left	5.8	21.7	0.048	10.0	19.9	0.047
7	Left	6.2	23.4	0.061	10.0	26.1	0.085
8	Left	5.7	24.5	0.060	10.0	22.6	0.087
9	Left	6.2	19.8	0.040	10.0	20.6	0.057
10	Left	6.2	22.6	0.057	10.0	20.6	0.058

Statistical analysis results

Geometric Coverage

Replacing the single-isocenter with a double-isocenter configuration increased prescription dose coverage along the trigeminal nerve from 6.2 ± 0.4 mm to 10.0 mm. Although the width of the 50% isodose line perpendicular to the nerve axis slightly decreased from 5.9 ± 0.3 mm to 5.6 ± 0.5 mm, it remained sufficient to encompass the nerve, which typically measures 2 mm to 3 mm in diameter. At higher isodose levels, particularly 80%, the width also narrowed from 3.8 ± 0.2 mm to 3.1 ± 0.2 mm, but this was effectively offset by an increase in axial coverage from 4.0 ± 0.2 mm to 6.2 ± 0.8 mm.

Maximum Dose to the Brainstem (Dmax)

The mean brainstem Dmax for the single-isocenter plan was 23.4±2.9 Gy (range, 19.8-29.6 Gy), compared to 22.5±3.3 Gy (range, 17.7-29.2 Gy) for the double-isocenter plan. The paired t-test showed no statistically significant difference between the two configurations (p=0.115), with a mean reduction of 0.9 Gy in Dmax, favoring the double-isocenter approach in most cases (7/10 patients).

Volume Receiving 10 Gy or Greater (V(10Gy))

The mean brainstem V(10Gy) for the single-isocenter plan was 0.054±0.011 cc (range, 0.040-0.077 cc), and 0.079±0.024 cc (range, 0.047-0.113 cc) for the double-isocenter plan. This difference was statistically significant (p<0.003), reflecting an average increase of 0.025 cc, though all values remained well below the 0.5 cc safety threshold.

## Discussion

With the intent to treat an extended segment of the trigeminal nerve in MS-TN, the double-isocenter configuration was employed. This dosimetric comparison of single-isocenter and double-isocenter gamma knife SRS plans assessed the feasibility of the approach in terms of potential toxicity management. The double-isocenter technique expanded the treated nerve segment from approximately 6 mm (conventional approach) to 10 mm, while maintaining the brainstem dose within safe limits at the same prescribed dose of 40 Gy to the 50% isodose line.

The analysis showed no statistically significant difference in Dmax to the brainstem between the two configurations (p=0.115), indicating that the double-isocenter technique did not compromise the critical goal of limiting the brainstem's maximum dose. While the double-isocenter plan resulted in a statistically significant increase in the volume of the brainstem receiving at least 10 Gy (V(10Gy), p<0.003), this increase was modest (from 0.054±0.011 cc to 0.079±0.024 cc), with no published data suggesting adverse clinical outcomes [18]. These findings align with established brainstem tolerance guidelines for SRS, where Dmax thresholds of <35 Gy and V(10Gy) <0.5 cc are recommended to minimize the risk of cranial neuropathy [18-20]. For instance, Milano et al.'s comprehensive review of single- and multifraction SRS dose-volume tolerances for the brain emphasizes that low-volume exposures (e.g., V(10Gy) <0.5 cc) are associated with <5-10% risk of symptomatic necrosis or neuropathy, supporting the clinical insignificance of the observed V(10Gy) increase in this study, as the absolute value of 0.079 cc remains well below their reported threshold [18]. Similarly, Sudahar et al.'s dosimetric analysis in CyberKnife SRS for TN reported comparable brainstem doses, with V(10Gy) values well below 0.5 cc correlating with low complication rates [19]. Timmerman's hypofractionation guidelines further corroborate that modest volume increases at intermediate doses (e.g., 10 Gy) are tolerable in ablative SRS, provided maximum doses remain constrained [20].

In the broader context of available literature on SRS for MS-TN, we hypothesize that expanding nerve coverage could address the limitations of conventional single-isocenter approaches, which often yield suboptimal long-term outcomes due to the diffuse demyelination characteristic of MS [2,7]. Retrospective studies, such as those by Helis et al. and Xu et al., report initial pain relief rates of 71% to 88% with single-isocenter GKRS, but with high recurrence (30% to 50%) and shorter pain-free intervals (median 1.1-4.6 years) compared to classical TN [7,4]. These poorer outcomes are attributed to multifocal plaques along the trigeminal pathway, which may not be adequately targeted by limited (4-6 mm) irradiation [2,3]. Recent studies on repeat GKRS, like those by Öztürk Özlük et al. and Samanci et al., demonstrate prolonged relief (median three to four years) with re-irradiation [11,12]. Because the isocenter for repeat SRS is typically placed some distance from the initial isocenter to avoid overlap, the overall irradiated nerve length after re-irradiation often approaches that of the double-isocenter approach. Thus, extending coverage to 10 mm in a single setting may better encompass demyelinating MS lesions, potentially improving efficacy as hypothesized. Prospective data from Tuleasca et al. support this, showing >80% initial response with GKRS but emphasizing the need for optimized targeting in MS cohorts [10].

Despite these promising findings, several limitations must be acknowledged. First, this is a retrospective dosimetric study with a small sample size (n=10). Although cases were selected to reflect anatomical variation encountered clinically, some unaccounted variability remains, which may limit generalizability. Second, toxicity feasibility was evaluated solely in terms of brainstem dose. Given the small and highly localized region of irradiation, other critical structures are not typically at risk, but the potential adverse effects on the trigeminal nerve itself due to extended coverage were not considered in this analysis. Currently, there is no volumetric dose model to predict neurotoxicity for the nerve in this context. While the cumulative length of irradiation resulting from initial and repeat gamma knife SRS may approximate that of a double-isocenter plan, it remains unclear whether this extended segment is responsible for the increased numbness following re-irradiation [11,12]. A more comprehensive set of metrics for evaluating toxicity is planned for the clinical trial, encompassing not only the brainstem but also a detailed assessment of nerve-specific toxicity and patient-reported outcomes.

Overall, our dosimetric feasibility supports advancing to clinical trials to evaluate whether targeted volume expansion could enhance durability. Although this study was conducted using the Leksell Gamma Knife SRS system, similar results are expected with other SRS systems that demonstrate proven capabilities for delivering steep dose gradients and high accuracy, such as CyberKnife or ZAP-X platforms, provided equivalent planning constraints are applied. In this study, we focused exclusively on potential brainstem toxicity, as it is the most critical organ at risk in TN SRS. While other structures may warrant consideration in broader radiotherapy planning, their exposure during gamma knife treatment is minimal due to the system’s highly conformal, isocentric design. In contrast, non-isocentric SRS systems, where non-isotropic beam arrangements may occur, require careful attention to avoid unnecessary exposure to other critical structures.

## Conclusions

This study demonstrates that the double-isocenter gamma knife SRS technique achieves its objective of expanding the treated nerve segment without compromising the safety profile for brainstem exposure, as Dmax remained equivalent, and the modest V(10Gy) increase was likely clinically negligible. These dosimetric findings support feasibility and lay the groundwork for clinical trials to evaluate efficacy and long-term outcomes in MS-TN patients. Future investigations should prioritize pain relief durability, recurrence rates, and comprehensive toxicity assessment to validate this novel approach.
